# Give It a Fair Trial

**Published:** 1889-02-09

**Authors:** 


### The Hospital

A Weekly Institutional Journal of

**Science, Medicine, Nursing, and Philanthropy.**

*For Hospitals, Asylums, Medical Practitioners, Students, Nurses, Families, Parishes Congregations, and the Charitable Public.*

Vol. V. No. 124.] [February 9, 1889.

### Give it a Fair Trial.

One of the most striking characteristics of a typical Englishman is his practicality. He has a clear eye for a fact. He can hardly ever be persuaded to settle concrete business by mere abstract discussion. He always likes to see how a thing will work in actual practice. This is a distinguishing merit and a sure guarantee of safe and steady progress. For a long time the roar of battle has been heard within the ranks of the medical profession in London. On the one side are ranged the general practitioners of medicine; on the other the hospitals. The practitioners are firmly convinced that the hospitals belong to a class which have always been obnoxious to the holders and lovers of vested interests. They believe and declare them to be "poachers"; poachers of a powerful, insidious, and dangerous character; poachers who have the rare good fortune to possess the sympathy of the philanthropic public, and a singular ability in extracting indefinite sums of money from the public pocket. The practitioners, in short, believe and declare that the London hospitals treat gratuitously thousands of people who can well afford to pay private medical attendants, and thus rob the struggling members of the profession to the extent of tens of thousands a-year. They affirm, moreover, that there is no kind of fairness in the competition between themselves and the hospitals, because the latter are backed by all the prestige of their splendid reputation, and all the resources of almost unlimited wealth.

Now this is a practical question, and must be treated practically. It is admitted on all hands that there are vast numbers of poor in the metropolis who need medical advice, but who can pay nothing at all for it. These are provided for, partly by the poor-law infirmaries, and partly by the general hospitals. The quarrel is not about them. The doctors do not want them, and indeed cannot afford to have them. Immediately above these is a class who can pay something. Philanthropists and hospital supporters declare that a large number of those who can pay a little, cannot pay sufficient to secure really skilled and efficient medical attention and nursing. But skilled medical attention and nursing are to this class among the very primest "necessaries of life." For illness to them means "no work," and "no work" means "no wages," and no wages means no support either for themselves or their families. It is clear, then, that when a working man is ill, nothing should be left undone which will restore him to health and work at the earliest possible moment. His own interests demand this with the utmost urgency, and the interests of his ratepaying neighbours, upon whom the burden of his family may fall whilst he is disabled, demand it with an equal insistance. What is to be done? Is the disabled workman in such a case to be left in his own poor home to get such medical attention, nursing, and feeding as he can, in order to give the general practitioner a chance of a picking, however small? Or is he to be taken in by the hospital, tended by skilled physicians, nursed by skilled nurses, fed from a bountiful and suitable table, and restored to health and work at the earliest possible moment? To ask these questions is to answer them; and to answer them in only one way. The hospital is the natural and proper refuge for those who cannot obtain at home adequate medical skill and competent nursing and care.

But now arises another question: Should the man who has been taken into the hospital, because he was unable to pay for adequate medical skill and competent nursing care at home, be asked or allowed to pay anything towards the support of the hospital? The answer of plain sense would be: "Yes, if he can." If, then, it be asked how much he is to be allowed to pay? The answer again will be: "As much as he can." So far it would seem there ought to be no difference of opinion. Even the general practitioners, if polled one by one, would admit that any little profit they might get out of sick workmen would not compensate them for the professional dissatisfaction they would feel in seeing cases protracted week after week by miserable surroundings and the incompetent nursing which alone the poor are able to obtain. Well, then, if this be so, is it not reasonable to think of providing hospitals in poor districts which shall always be ready to receive those of the working class who cannot pay for adequate medical skill and nursing care at home? And is it not reasonable to ask working men to help to support such hospitals? They cannot support them when they are ill! Then, of course, they must support them when they are well. They cannot support them with large sums! Then they must support them with small sums. But the small sums will be worth nothing unless they are frequent and regular! Then they must be frequent and regular: and this is the "Provident System."

Thus far the appeal has been to reason, right feeling, and the stern logic of necessity. We have often maintained a flood of light would be thrown on the whole question if a hospital in a working-class district would try to work out the difficult problem of medical relief to the industrious but necessitous workman on the "Provident" lines here indicated! As a matter of fact, this is now being done; and all reasonable philanthropists and medical men are looking on with the utmost interest to see how these principles work in actual operation.

Sir Edmund Currie, a competent and reasonable man, a proved friend of the industrial classes, a respecter of the interests of medical practitioners, a man who wishes to do right and right only, has induced the authorities and the medical staff of the Metropolitan Hospital, in the Kingsland Road, to try the Provident System. He has done the thing openly, and by mere force of argument and conviction. He sprang no mine on either the medical staff, the supporters of the hospital, or the practitioners in the neighbourhood, whose interests might possibly be affected. He gave them each and all the fullest opportunities of meeting him in debate, of refuting him in argument. The neighbouring practitioners, it is said, left Sir Edmund severely alone. Whilst the matter was *sub judice* no practitioner seems to have made any protest, or otherwise indicated his dissatisfaction. It seemed, therefore, as if the experiment were to be made under a clear sky, and the results were to be dispassionately observed and considered for future use and guidance.

But that hope is dashed to the ground. The practitioners of the neighbourhood are now up in arms. They have met, and they have protested, and their protest has been printed by a contemporary. The protest amounts to this, that the Provident Hospital in the Kingsland Road will injuriously affect the pecuniary interests of the private medical practitioners in the neighbourhood. To this Sir Edmund Currie says, "No." The hospital does not seek those who can pay private practitioners. It fixes a wage limit, and takes no members who are above that limit. The *Lancet*, adopting a principle of the narrowest opportunism, supports the practitioners in their protest, and in spite of the wage limit, gives its verdict against Sir Edmund Currie.

On these facts we make an appeal to all dispassionate readers, both medical and lay. The medical practitioners, discussing the question from an interested standpoint, affirm that the "provident system" of hospital management is bad in itself and injurious to them. Sir Edmund Currie and his sympathisers, looking at it from a public point of view, say it is good in itself, and will prove not only not injurious, but pecuniarily advantageous to the general practitioner. Who is to decide between them? It is impossible to settle the question by mere discussion. Every reasonable man, both lay and medical, will at once say, *Fiat experimentum:* Let the system be tried. The present methods of management at other London hospitals give no satisfaction to outside medical attendants. They are only less hateful to them than the Provident System, because the extent of the injury they do to private practice is partly understood, whereas that which might be caused by the provident system is uncertain, and therefore is the more feared. But how do doctors know that the provident system might not largely or entirely cure the very evils they complain of in the present methods of hospital administration? We repeat, "Let the thing be tried." It must be tried somewhere, and there is no more suitable neighbourhood in the whole of London than that in which the Metropolitan Hospital is situated, in the Kingsland Road. There is not in all the kingdom a more competent man than Sir Edmund Currie to take the lead in such a forward movement. We counsel medical practitioners not to cry out before they are hurt, but to await with calmness and self-respecting dignity the results of the only process which can ever be conclusive and final.

